# Predictive impact of sarcopenia in solid cancers treated with immune checkpoint inhibitors: a meta‐analysis

**DOI:** 10.1002/jcsm.12755

**Published:** 2021-08-01

**Authors:** Yukinori Takenaka, Ryohei Oya, Norihiko Takemoto, Hidenori Inohara

**Affiliations:** ^1^ Department of Otorhinolaryngology Kansai Medical Hospital Osaka Toyonaka Japan; ^2^ Department of Otorhinolaryngology‐Head and Neck Surgery Osaka University Graduate School of Medicine Suita Osaka Japan

**Keywords:** Immune checkpoint inhibitor, Sarcopenia, Solid cancer, Non‐small cell lung cancer, Melanoma

## Abstract

Sarcopenia, which is characterized by a decrease in muscle quantity or quality, is commonly observed in patients with cancer. Recent research has reported contradictory results on the association between sarcopenia and the efficacy of immune checkpoint inhibitors (ICIs). We conducted a systematic review and meta‐analysis to investigate this discrepancy. We systematically searched three electronic databases to identify articles reporting on the association between sarcopenia and treatment outcomes in patients with solid cancers who received ICIs. The outcomes assessed were hazard ratios (HRs) for overall survival (OS) and progression‐free survival (PFS), and odds ratios (ORs) for objective response rate (ORR), disease control rate (DCR), and toxicity. Pooled estimates and their 95% confidence intervals (CIs) were calculated. A total of 2501 patients from 26 studies were analysed. Sarcopenia was observed in 44.7% (95% CI: 38.2–51.3) of the patients and was significantly associated with poor survival (HR = 1.55, 95% CI = 1.32–1.82 for OS and HR = 1.61, 95% CI = 1.35 to 1.93 for PFS). The HRs (95% CIs) for OS according to the diagnostic measures used were 1.97 (0.88–4.41) for psoas muscle index (PMI), 1.41 (0.87–2.28) for skeletal muscle density (SMD), and 1.43 (1.23–1.67) for skeletal mass index (SMI). The HRs (95% CIs) for PFS were 1.86 (1.08–3.21) for PMI, 1.27 (0.94–1.71) for SMD, and 1.38 (1.11–1.71) for SMI. Poor radiological response to ICI therapy was observed in patients with sarcopenia (OR = 0.52, 95% CI = 0.34–0.80 for ORR and OR = 0.45, 95% CI = 0.30–0.67 for DCR). The ORs for ORR (95% CIs) were 0.56 (0.15–2.05) for PMI and 0.78 (0.56–1.09) for SMI. The oncologic outcomes associated with melanoma and non‐small cell lung cancer (NSCLC) were comparable with those observed overall (HR for OS = 2.02, 95% CI = 1.26–3.24 for melanoma and HR for OS = 1.61, 95% CI = 1.19–2.18 for NSCLC). In contrast, the occurrence of severe toxicity was not associated with sarcopenia (OR = 1.13, 95% CI = 0.51–2.52). Poor survival and poor response in patients with sarcopenia indicate a negative association between sarcopenia and efficacy of ICIs. Sarcopenia's predictive ability is consistent across various tumour types. For the selection of patients who may respond to ICIs pre‐therapeutically, the presence of sarcopenia should be assessed in clinical practice.

## 1 Background

Surgery, radiation, and chemotherapy have been the three main pillars of cancer treatment for decades. However, recent rapid progress in immunotherapy has changed this paradigm.^1^ Immune checkpoint inhibitor (ICI) therapy is the most frequently used immunotherapy against various cancer types. ICIs are predominantly used for the treatment of recurrent and metastatic diseases that cannot be cured with conventional therapy; however, the indications for their use have been expanding.^2^ The use of ICIs can significantly lengthen survival and sometimes result in a long duration of disease control even in patients with advanced disease and disease progression. So far, seven drugs—atezolizumab, avelumab, cemiplimab, durvalumab, ipilimumab, nivolumab, and pembrolizumab—have been approved for use in clinical practice. Although their clinical benefit is apparent, the use of ICIs is limited owing to the associated cost. To identify patients who may benefit the most from ICIs, companion and complementary diagnostics have been developed.^3^ All ICIs, except ipilimumab, inhibit the binding between programmed death protein 1 (PD‐1) and programmed death ligand 1 (PD‐L1). Therefore, the immunohistochemical measurement of PD‐L1 expression is employed as a tool for companion diagnostics.^2^ However, partly owing to the heterogeneous PD‐L1 expression in tumour tissues, its predictive ability is not satisfactory for use in clinical practice.^4^ Other cancer immunity‐associated biomarkers used for companion diagnostics include tumour mutation burden and microsatellite instability.^3^ However, when used alone, these biomarkers have limited predictive value. Efforts are underway for the identification of other biomarkers.^5^

Sarcopenia is a skeletal muscle disorder characterized by reduced muscle strength and muscle quantity.^6^ Recently, a meta‐analysis of various types of cancers demonstrated an association between sarcopenia and prognoses.^7^ In addition, an increasing number of studies are focusing on the impact of sarcopenia on ICI treatment efficacy.^8^, ^9^, ^10^, ^11^, ^12^, ^13^, ^14^, ^15^, ^16^, ^17^, ^18^, ^19^, ^20^, ^21^, ^22^, ^23^, ^24^, ^25^, ^26^, ^27^, ^28^, ^29^, ^30^, ^31^, ^32^, ^33^ However, most previous studies on the topic had a retrospective design and included a small number of patients in whom various methods were employed for the diagnosis of sarcopenia. Therefore, the predictive value of sarcopenia in ICI therapy requires elucidation.

Meta‐analyses have advantages in that they can generate a pooled effect size, as deduced from the results of previous studies and thus can yield more reliable conclusions using data from a larger number of patients. This study aimed to investigate, using a meta‐analysis, whether sarcopenia status is predictive of oncologic outcomes in patients treated with ICIs. Further, we also sought to determine the differences between various tools and tests for sarcopenia in the prediction of prognoses.

## 2 Methods

### 2.1 Search strategy

This study was conducted in accordance with Preferred Reporting Items for Systematic Reviews and Meta‐Analyses guidelines.^34^ We conducted a search for published studies focusing on the association between sarcopenia and ICI efficacy in the following electronic databases: PubMed www.ncbi.nlm.nih.gov/pubmed, Scopus www.elsevier.com/online‐tools/scopus, and Ichushi*‐*Web https://search.jamas.or.jp, which contains bibliographic information and abstracts of articles in Japanese journals (Japan Medical Abstracts Society) from inception to 4 May 2021. The search terms were (i) ‘CTLA‐4’ or ‘CTLA4’ or ‘cytotoxic T‐lymphocyte‐associated protein 4’ or ‘CD152’ or ‘PD‐1’ or ‘PD1’ or ‘programmed cell death protein 1’ or ‘CD279’ or ‘PD‐L1’ or ‘PDL1’ or ‘programmed death‐ligand 1’ or ‘CD274’ or ‘atezolizumab’ or ‘avelumab’ or ‘cemiplimab’ or ‘durvalumab’ or ‘ipilimumab’ or ‘nivolumab’ or ‘pembrolizumab’ and (ii) ‘sarcopenia’ or ‘sarcopenic’ or ‘muscle index’ or ‘muscle mass’ or ‘muscle depletion’ or ‘muscular atrophy’ or ‘muscle strength’ or ‘muscle quality’ or ‘muscle quantity’. The references in the retrieved articles were manually searched for associated studies.

### 2.2 Study selection

Articles in English or Japanese that met the following criteria were included in this study: (i) patients: patients with solid cancers treated with ICIs; (ii) exposure: sarcopenia was defined based on the diagnostic modalities recommended by consensus statements^6^, ^35^; (iii) comparison: non‐sarcopenia group; and (iv) outcome: overall survival (OS), progression‐free survival (PFS), objective response rate (ORR), and disease control rate (DCR), as defined by response evaluation criteria in solid tumours^36^ and ICI‐induced toxicity. The exclusion criteria were as follows: (i) study design: animal study, review, case reports, and conference abstracts; (ii) articles written in languages other than English or Japanese; (iii) the hazard ratio (HR) or odds ratio (OR) for outcomes were neither described in the manuscript nor estimated from the published data. Two of the authors (Y. T. and R. O.) independently evaluated the electronically searched titles. All potentially relevant publications were retrieved. Disagreements were resolved by consensus.

### 2.3 Data extraction

The following data were extracted: name of first author, year of publication, institution and country, number of patients, number of outcomes according to sarcopenia status, disease stage, ICI drug names, toxicity, diagnostic measures for sarcopenia and their cut‐off methods and cut‐off values, and HRs and ORs and their 95% confidence intervals (CIs). The HRs, ORs, and 95% CIs were extracted preferentially from multivariate or univariate analyses. When HRs were not provided in the manuscript, survival data were extracted from Kaplan–Meier curves and estimated using the method proposed by Tierney *et al*.^37^ The Newcastle–Ottawa Scale^38^ was used to assess the quality of the included studies; those with a score ≥6 were considered high‐quality studies.

### 2.4 Statistical analysis

Pooled HRs, ORs, and their 95% CIs were estimated with both a random effect model and a fixed effect model using Comprehensive Meta‐Analysis Version 2 (Biostat, Englewood, NJ, USA). First, we investigated the predictive impact of sarcopenia on OS, PFS, objective response, disease control, and toxicity. The mean HR was used as the representative of the study in a meta‐analysis when more than one diagnostic procedure for sarcopenia was used.^12^, ^16^, ^25^, ^26^, ^29^ Second, we conducted meta‐analyses according to each diagnostic procedure. Sensitivity analyses were performed by the sequential omission of each individual study. Subgroup analyses were conducted for primary tumour sites and ICIs. Publication bias was assessed using the funnel plot and tested with Egger's regression intercept test. Heterogeneity was assessed using Cochran's *Q* test and *I* ^2^ statistics. All statistical tests were two‐sided, and significance was defined by a *P*‐value <0.05. The included studies differed in the tumour sites, prior treatment, ICIs used, institutions, and diagnostic measures for sarcopenia and their cut‐off values. Owing to the heterogeneity among the studies, a random effect model was preferred in this manuscript.

The protocol for this meta‐analysis is available in UMIN (registration code: UMIN000042621).

## 3 Results

### 3.1 Literature search results

**Figure 1:**
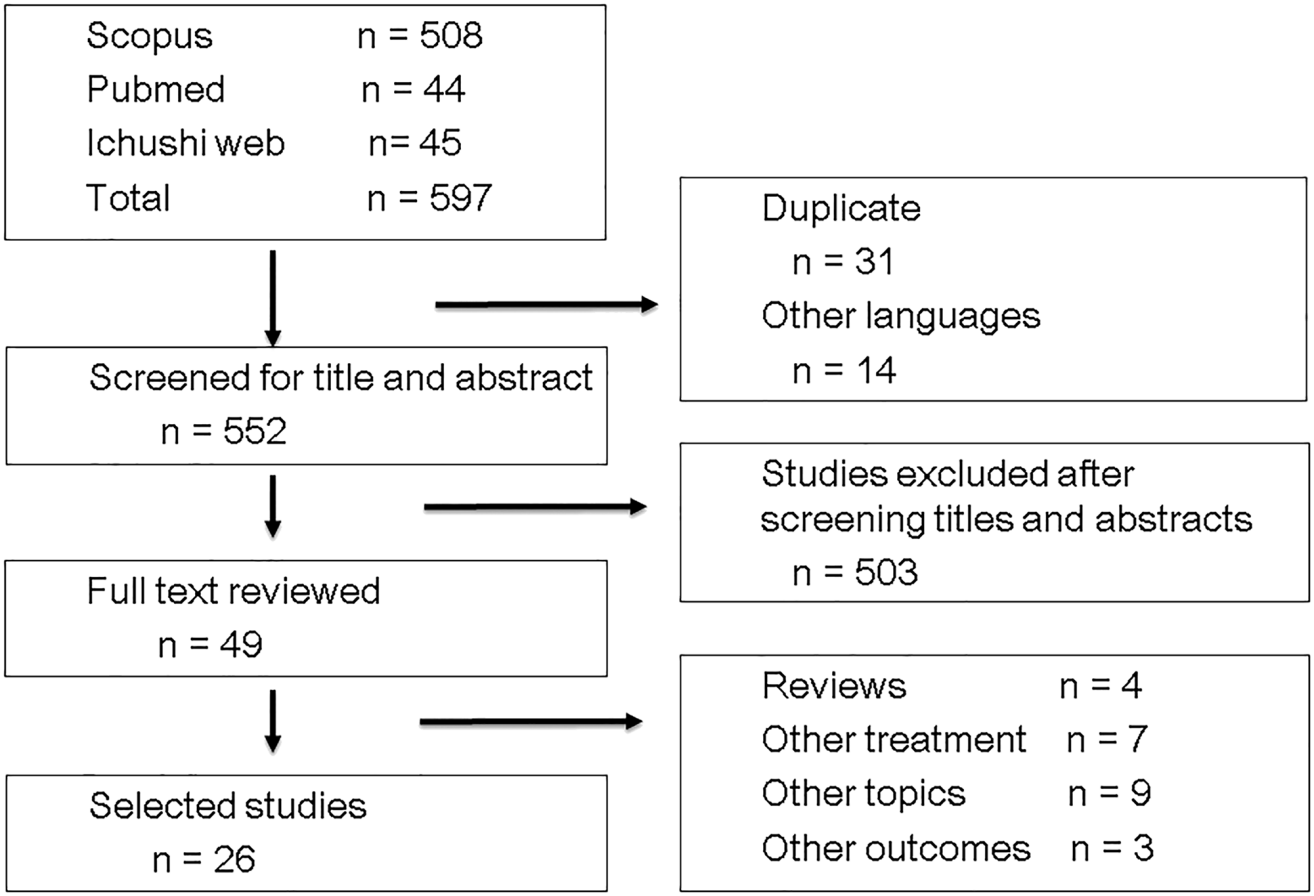
Flow diagram of article selection.

The electronic database search for articles from the inception of each database to 4 May 2021 led to the retrieval of 597 records (*Figure* 1). We excluded duplicate entries and articles written in languages other than English and Japanese and then screened for titles and abstracts. The full texts of the 49 studies selected were then inspected according to the inclusion and exclusion criteria; finally, 26 studies^8^, ^9^, ^10^, ^11^, ^12^, ^13^, ^14^, ^15^, ^16^, ^17^, ^18^, ^19^, ^20^, ^21^, ^22^, ^23^, ^24^, ^25^, ^26^, ^27^, ^28^, ^29^, ^30^, ^31^, ^32^, ^33^ comprising 2501 patients were included in the systematic review. Two studies by Cortellini *et al*. contain overlapping data.^11^, ^16^ Newer and more detailed data were used when the same outcome data were provided in both studies. All 26 articles were written in English.

### 3.2 Diagnosis and prevalence of sarcopenia

*Table* 1 shows the characteristics of the included studies. Nine studies each were conducted in Europe and Japan, and three were performed in the USA. All the studies used computed tomography (CT) as a modality to diagnose sarcopenia. None of the included studies used questionnaires, dual‐energy X‐ray absorptiometry (DXA), or bioelectrical impedance assay (BIA). Of the diagnostic methods, the skeletal mass index (SMI) was the most commonly used,^9^, ^10^, ^11^, ^16^, ^18^, ^19^, ^22^, ^23^, ^25^, ^26^, ^27^, ^29^, ^30^, ^31^, ^33^ followed by the psoas muscle index (PMI)^14^, ^17^, ^20^, ^21^, ^24^, ^26^, ^28^ and skeletal muscle density (SMD).^15^, ^16^, ^29^ Of the 15 articles that employed SMI, five^10^, ^11^, ^18^, ^19^, ^29^ used the cut‐off value described by Martin e*t al*.,^39^ while of the seven that employed PMI, four^14^, ^24^, ^26^, ^28^ used the cut‐off value for Asian adults.^40^ The prevalence of sarcopenia ranged from 21.9% to 75.0%, and the pooled prevalence of sarcopenia was 44.7% (95% CI: 38.2–51.3) (Supporting Information, *Figure* S1).

**Table 1:** Study characteristics

Year	Author	Country	Site	Treatment	Diagnostic method	Cut‐off value	Outcome	No. of patients	Age median [range] {interquartile range} mean ± SD	Gender (male/female)	Newcastle–Ottawa scale
2015	Sabel	USA	Melanoma	Ipilimumab	Psoas density	Highest quartile	DCR, ORR, and OS	44	55.1 [15–90]	84/49	4
2016	Dercle	France	Melanoma, lung cancer, bladder cancer, RCC	Anti‐PD1 and anti‐PDL1	SMI	53	OS	251	56 ± 13	131/120	7
2017	Daly	Ireland	Melanoma	Ipilimumab	Muscle loss at L3	7.5% (lowest quartile)	OS	84	54 [22–85]	52/32	7
					SMI	Male, 43 for BMI < 25, 53 for BMI ≥ 25; Female, 41	Toxicity				
2019	Cortellini	Italy	NSCLC	Nivolumab	SMI	Male, 43 for BMI < 25, 53 for BMI ≥ 25; Female, 41	ORR and toxicity	22	67 [41–82]	18/5	4
2019	Deike‐Hofmann	Germany	Melanoma	Ipilimumab	Mean psoas density	45 (lower quartile)	PFS	147	60 {49.5–66.5}	90/57	7
2019	Nishioka	Japan	NSCLC	Nivolumab and pemblolizumab	Decrease of the psoas major muscle area	10%	DCR, ORR, and PFS	38	68.7 [46–85]	26/12	4
2019	Shiroyama	Japan	NSCLC	Nivolumab and pemblolizumab	PMI	Male, 6.36; Female, 3.92	DCR, ORR, and PFS	42	Sarcopenia group: 72 [51–87]; Non‐sarcopenia group: 69 [37–78]	26/16	6
2020	Chu	Canada	Melanoma	Ipilimumab	SMD	BMI > 25; 20 HU; BMI < 25; 42	DCR, ORR, OS, PFS, and toxicity	97	56 [25–91]	58/39	6
2020	Cortellini	Italy	NSCLC, melanoma, RCC, and others	Atezolizumab, nivolumab, pemblolizumab, and others	SMD	Male, 24.2 for BMI < 25; 35.6 for BMII ≥ 25; Female, 27.9 for BMI < 25, 37.4 for BMII ≥ 25	ORR, OS, and PFS	100	66 [25–88]	67/33	
					SMI	Male, 48.4 for BMI < 25, 50.2 for BMII ≥ 25; Female, 36.9 for BMI < 25, 59.6 for BMII ≥ 25	ORR, OS, and PFS				5
2020	Crombe	France	Metastatic solid cancers	Anti‐PD1, anti‐PDL1, and anti‐PDL1/CTLA4	PMI decrease	Lowest tertile	PFS	117	63 [33.9–84.3]	62/55	6
2020	Fukushima	Japan	Urothelial carcinoma	Pemblolizumab	SMI	Male, 43 for BMI < 25, 53 for BMI ≥ 25; Female, 41	ORR, OS, PFS, and toxicity	28	74 [70–82]	19/9	5
2020	Hirsch	France	Solid cancer	Nivolumab	SMI	Male, 43 for BMI < 25, 53 for BMI ≥ 25; Female, 41	Toxicity	87	N/A	N/A	8
2020	Hu	USA	Melanoma	Pemblolizumab	PMI	Bottom tertile	ORR and toxicity	156	66 [21–93]	91/65	5
2020	Kano	Japan	Gastric cancer	Nivolumab	PMI	Male, 3.6; Female, 2.9	DCR, ORR, PFS, and toxicity	31	70 [35–83]	21/10	5
2020	Kim N	Korea	HCC	Nivolumab	SMI	Male, 42; Female, 38	DCR, ORR, PFS, and OS	102	61.3 [54–69]	87/15	7
2020	Kim Y	Korea	Gastric cancer	Pembrolizumab and nivolumab	SMI	Male, 49; Female, 31	DCR, ORR, OS, and PFS	147	57.0 ± 12.3	93/54	8
2020	Minami	Japan	NSCLC	Nivolumab, pemblolizumab, and atezolizumab	PMI	Male, 6.36; Female, 3.92	DCR, ORR, OS, and PFS	74	Sarcopenia group: 69 {63–74}; non‐sarcopenia group: 70 {61–73}	48/26	7
2020	Roch	France	NSCLC	Nivolumab and pemblolizumab	SMI decrease	5%	DCR, PFS, and OS	142	63.54 ± 10.58	93/49	5
					SMI	Male, 52.4; Female, 38.5	DCR, PFS, and OS				
2020	Shimizu	Japan	Urothelial carcinoma	Pembrolizumab	PMI	Male, 6.36; Female, 3.92	OS, PFS, and toxicity	27	73 [52–82]	23/4	5
					PMI decrease(1 month from baseline)	5%	OS and PFS				
					SMI decrease	5%	OS and PFS				
2020	Takada	Japan	NSCLC	Nivolumab and pemblolizumab	SMI	Male, 25.63; Female, 21.73	DCR, ORR, OS, and PFS	103	67 [36–88]	84/19	5
2020	Tsukagoshi	Japan	NSCLC	Nivolumab	PMI	Male, 6.36; Female, 3.92	DCR, ORR, OS, and PFS	30	67 [47–82]	23/7	6
2020	Young	USA	Melanoma	Ipilimumab + nivolumab, pembrolizumab, nivolumab, and atezolizumab	SMD	41 for BMI < 25, 33 for BMI ≥ 25	ORR, OS, and PFS	287	63 [20–89]; 61 ± 14.4	184/103	7
					SMG	1475	ORR, OS, and PFS				
					SMI	Male, 43 for BMI < 25, 53 for BMI ≥ 25; Female, 41	ORR, OS, and PFS				
2021	Akce	Canada	HCC	Anti‐PD‐1 antibody	SMI	Male, 43; Female, 39	OS and PFS	57	Median 66	44/13	5
2021	Loosen	Germany	NSCLC, melanoma, urothelial cancer, GI cancer, head and neck cancer, and others	Nivolumab, pembrolizumab, nivolumab + ipilimumab, and others	SMD decrease	−0.4	OS	88	67 [34–87]	58/30	6
					SMI decrease	−6.18					
2021	Nishioka	Japan	NSCLC	Nivolumab, pembrolizumab, and atezolizumab	SMD	33 for BMI ≧ 25, 41 for BMI < 25	ORR, OS, and PFS	156	67 [33–85]	101/55	7
					SMI	Male, 53 for BMI ≧ 25, 43 for BMI < 25; Female, 41					
2021	Youn	Canada	Melanoma	Nivolumab or nivolumab + ipilimumab	SMD	25.65	OS	44	57 [29–79]	25/19	6

DCR, disease control rate; GI, gastrointestinal; HCC, hepatocellular carcinoma; NSCLC, non‐small cell lung cancer; N/A, not available; ORR, objective response rate; OS, overall survival; PFS, progression‐free survival; PMI, psoas muscle index; SMD, skeletal muscle density; SMG, skeletal muscle gauge; SMI, skeletal muscle index.

### 3.3 Overall survival and sarcopenia

**Figure 2:**
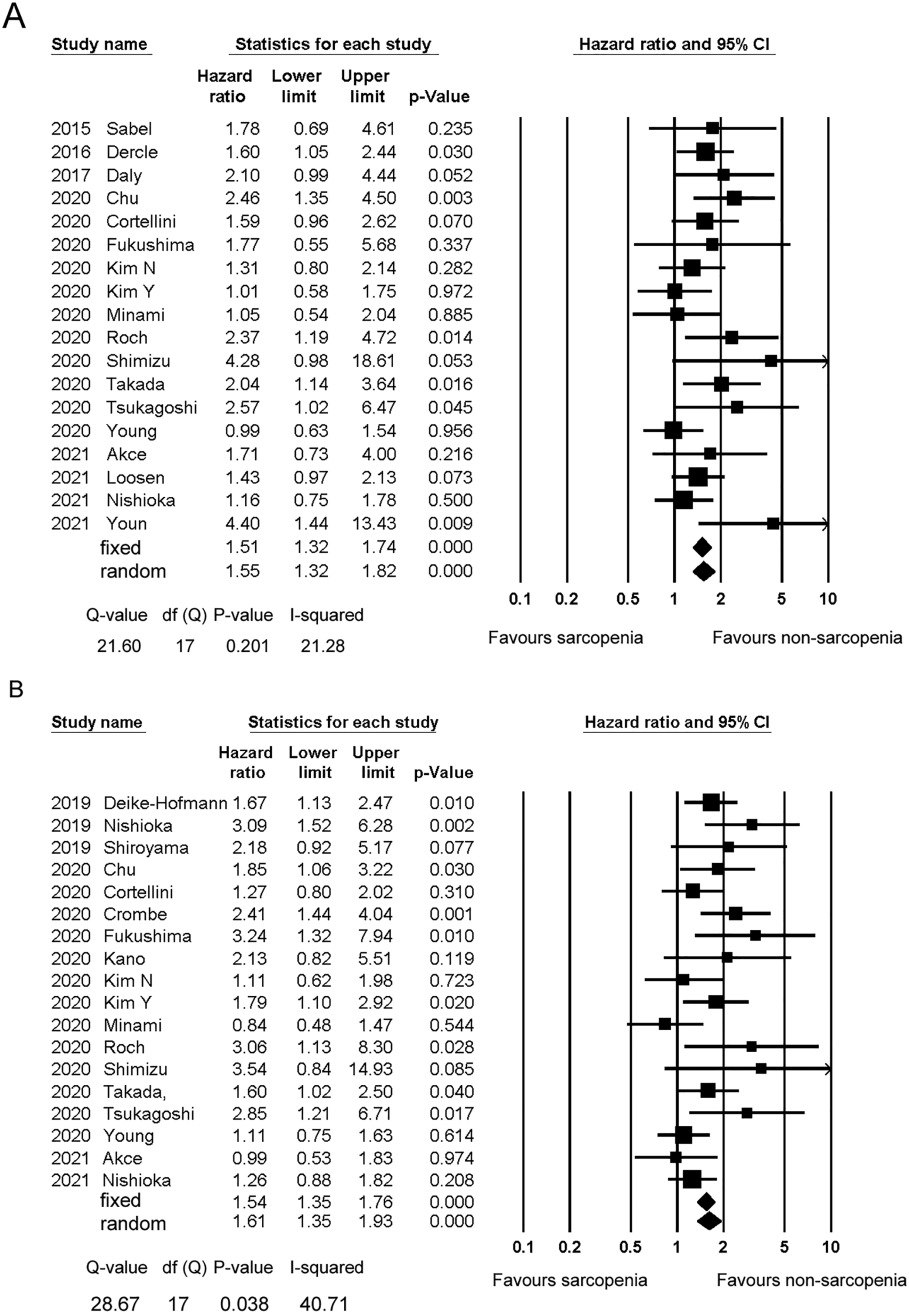
Forest plot showing the hazard ratios for overall survival *(A)* and progression‐free survival *(B)* between the sarcopenia and non‐sarcopenia patients. The squares represent the hazard ratios for each study. The sizes of the squares and the horizontal lines crossing the squares represent the weight of the study in the random effect model and the 95% confidence intervals, respectively.

Eighteen studies investigated the association between sarcopenia and OS.^8^, ^13^, ^14^, ^15^, ^16^, ^18^, ^20^, ^21^, ^22^, ^23^, ^24^, ^27^, ^28^, ^29^, ^30^, ^31^, ^32^, ^33^ The HRs for OS ranged from 0.76 to 6.21. Multivariate analyses were performed in 13 studies.^9^, ^10^, ^15^, ^16^, ^22^, ^24^, ^25^, ^26^, ^27^, ^29^, ^30^, ^32^, ^33^ HRs were estimated using the Kaplan–Meier curve in three studies.^8^, ^18^, ^31^ The meta‐analysis demonstrated the significant predictive ability of sarcopenia for OS (HR [95% CI] 1.55 [1.32–1.82]) (*Figure* 2A). The results of the sensitivity analysis are shown in the Supporting Information, *Table* S1.

The HRs for OS according to the diagnostic measures used are shown in the Supporting Information, *Table* S2. PMI, SMD, and SMI were employed for dichotomization in three,^24^, ^26^, ^28^ five,^15^, ^16^, ^29^, ^31^, ^32^ and 10 studies,^9^, ^16^, ^18^, ^22^, ^23^, ^25^, ^27^, ^29^, ^31^, ^33^ respectively. The HRs (95% CIs) were 1.97 (0.88–4.41) for PMI, 1.41 (0.87–2.28) for SMD, and 1.43 (1.23–1.67) for SMI. There were no significant differences among the different diagnostic measures (*P* = 0.507).

### 3.4 Progression‐free survival and sarcopenia

Eighteen studies^12^, ^13^, ^14^, ^15^, ^16^, ^17^, ^18^, ^21^, ^22^, ^23^, ^24^, ^25^, ^26^, ^27^, ^28^, ^29^, ^31^, ^33^ investigated the association between sarcopenia and PFS. Multivariate analysis was performed in 14 studies.^14^, ^15^, ^16^, ^17^, ^22^, ^23^, ^24^, ^25^, ^26^, ^27^, ^28^, ^29^, ^31^, ^33^ The HRs for PFS were estimated using the Kaplan–Meier curve analysis in two studies.^13^, ^21^ The HRs for PFS ranged from 0.84 to 12.80. Sarcopenia was significantly associated with worse PFS values (random effect model, HR [95% CI] 1.61 [1.35–1.93]) (*Figure* 2B). The results of the sensitivity analysis are shown in the Supporting Information, *Table* S3. The result was similar when any individual study was removed from the analysis.

The HRs for PFS according to the diagnostic measures employed are shown in the Supporting Information, *Table* S4. PMI, SMD, and SMI were employed for dichotomization in five,^14^, ^21^, ^24^, ^26^, ^28^ four,^15^, ^16^, ^29^, ^31^ and nine studies,^16^, ^18^, ^22^, ^23^, ^25^, ^27^, ^29^, ^31^, ^33^ respectively. SMI and PMI were predictors of PFS (HR = 1.38, 95% CI = 1.11–1.71; and HR = 1.86, 95% CI = 1.08–3.21, respectively). In contrast, SMD was not associated with PFS (HR = 1.27, 95% CI = 0.94–1.71). There were no significant differences among the different diagnostic measures (*P* = 0.207).

### 3.5 Objective response and sarcopenia

**Figure 3:**
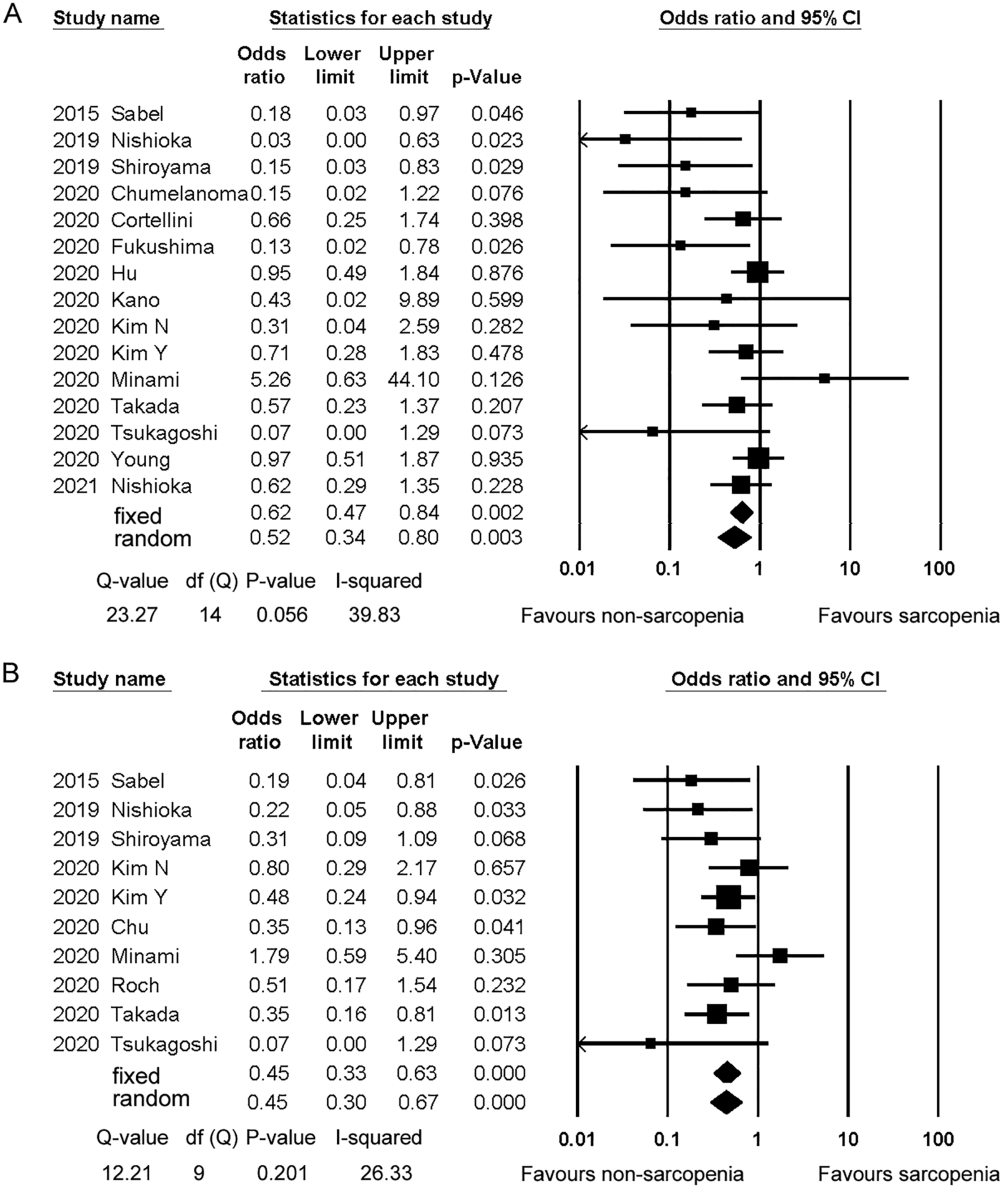
Forest plot showing the odds ratios for objective response rate *(A)* and disease control rate *(B)* between the sarcopenia and non‐sarcopenia patients. The squares represent the hazard ratios for each study. The sizes of the squares and the horizontal lines crossing the squares represent the weight of the study in the random effect model and the 95% confidence intervals, respectively.

Objective response rate was investigated in 15 studies.^8^, ^13^, ^14^, ^15^, ^16^, ^18^, ^20^, ^21^, ^22^, ^23^, ^24^, ^27^, ^28^, ^29^, ^31^ Only one study used multivariate analyses.^29^ The ORs for ORR ranged from 0.03 to 5.26. Sarcopenia was significantly associated with worse response (OR = 0.52, 95% CI = 0.34–0.80) (*Figure* 3A). The results of the sensitivity analysis are shown in the Supporting Information, *Table* S5. The result was similar when any individual study was removed from the analysis.

PMI, SMD, and SMI were employed for dichotomization in five,^14^, ^20^, ^21^, ^24^, ^28^ four,^15^, ^16^, ^29^, ^31^ and seven studies,^16^, ^18^, ^22^, ^23^, ^27^, ^29^, ^31^ respectively. The ORs for each procedure showed a tendency for worse response in sarcopenia patients. The pooled ORs (95% CIs) were 0.56 (0.15–2.05) for PMI, 0.51 (0.22–1.17) for SMD, and 0.78 (0.56–1.09) for SMI (Supporting Information, *Table* S6). There were no significant differences among the different diagnostic measures (*P* = 0.153). The ORs and 95% CIs for other diagnostic procedures are also shown in the Supporting Information, *Table* S6.

### 3.6 Disease control and sarcopenia

Disease control rate was investigated in 10 studies.^8^, ^13^, ^14^, ^15^, ^22^, ^23^, ^24^, ^25^, ^27^, ^28^ None of the 10 studies performed multivariate analyses for DCR. The ORs for DCR ranged from 0.07 to 1.79. The pooled OR (95% CI) in the 10 studies was 0.45 (0.30–0.67) (*Figure* 3B). Although the studies by Minami and Tsukagoshi seemed to be outliers, the exclusion of either study did not change the results significantly (Supporting Information, *Table* S7).

Psoas muscle index and SMI were employed for dichotomization in three studies each^14^, ^24^, ^28^.^22^, ^25^, ^27^ The pooled ORs (95% CIs) were 0.47 (0.09–2.52) for PMI and 0.51 (0.34–0.78) for SMI (Supporting Information, *Table* S8). There were no significant differences among the different diagnostic measures (*P* = 0.754).

### 3.7 Subgroup analysis

Subgroup analyses using a random effect model were performed according to the primary tumour site (*Table* 2). Melanoma and non‐small cell lung cancer (NSCLC) were the most commonly investigated tumours; other tumours were included only in two or fewer studies. The pooled HRs and ORs for melanoma and NSCLC showed a statistically significant association between sarcopenia and worse OS, worse PFS, and worse DCR. Similar results were obtained with other types of tumours, although some failed to show a significant result.

**Table 2:** Hazard ratios and odds ratios according to the primary tumour site

	No. of studies	No. of patients	Estimates	Lower limit	Upper limit	*P‐value*
OS			HR			
Gastric cancer	1	149	1.01	0.58	1.75	0.972
HCC	2	159	1.40	0.91	2.14	0.121
Melanoma	6	583	2.02	1.26	3.24	0.003
NSCLC	6	551	1.61	1.19	2.18	0.002
Urothelial cancer	2	55	2.49	1.00	6.20	0.051
PFS			HR			
Gastric cancer	2	180	1.86	1.20	2.87	0.005
HCC	2	159	1.05	0.69	1.60	0.813
Melanoma	4	558	1.53	1.13	2.07	0.006
NSCLC	8	631	1.69	1.24	2.31	0.001
Urothelial cancer	2	55	3.32	1.55	7.11	0.002
ORR			OR			
Gastric cancer	2	178	0.68	0.27	1.69	0.406
HCC	1	102	0.31	0.04	2.59	0.282
Melanoma	4	584	0.63	0.30	1.31	0.295
NSCLC	7	465	0.49	0.20	1.22	0.127
Urothelial cancer	1	28	0.13	0.02	0.78	0.026
DCR			OR			
Gastric cancer	1	147	0.48	0.24	0.94	0.032
HCC	1	102	0.80	0.29	2.17	0.657
Melanoma	2	141	0.28	0.12	0.66	0.003
NSCLC	6	429	0.43	0.22	0.87	0.019

CI, confidence interval; DCR, disease control rate; HCC, hepatocellular carcinoma; HR, hazard ratio; NSCLC, non‐small cell lung cancer; OR, odds ratio; ORR, objective response rate; OS, overall survival; PFS; progression‐free survival.

Next, we conducted a subgroup analysis for the ICI drugs (*Table* 3). Data on ICI monotherapy were investigated in four studies on Ipilimumab,^8^, ^10^, ^12^, ^15^ five on Nivolumab,^11^, ^19^, ^21^, ^23^, ^28^ and three on pembrolizumab.^18^, ^20^, ^26^ HR for OS and PFS, OR for ORR, and DCR favoured non‐sarcopenia in all drugs. The difference among the drugs was not significant with respect to any outcomes (*P* = 0.670 for OS, *P* = 0.291 for PFS, *P* = 0.107 for ORR, and *P* = 0.876 for DCR).

**Table 3:** Hazard ratios and odds ratios according to immune checkpoint inhibitors

	No. of studies	No. of patients	Estimates	95% CI		*P‐value*
				Lower limit	Upper limit	
OS			HR			
Ipilimumab	3	225	2.20	1.44	3.35	0.000
Nivolumab	2	132	1.63	0.88	3.03	0.121
Pembrolizumab	2	55	2.49	1.00	6.20	0.051
PFS			HR			
Ipilimumab	2	244	1.73	1.25	2.38	0.001
Nivolumab	3	163	1.74	0.95	3.20	0.072
Pembrolizumab	2	55	3.32	1.55	7.11	0.002
ORR			OR			
Ipilimumab	2	141	0.16	0.04	0.62	0.008
Nivolumab	4	185	0.44	0.11	1.72	0.239
Pembrolizumab	2	184	0.43	0.06	2.82	0.375
DCR			OR			
Ipilimumab	2	141	0.28	0.12	0.66	0.003
Nivolumab	2	132	0.34	0.03	3.48	0.367

CI, confidence interval; DCR, disease control rate; HR, hazard ratio; OR, odds ratio; ORR, objective response rate.

### 3.8 Severe toxicity and sarcopenia

**Figure 4:**
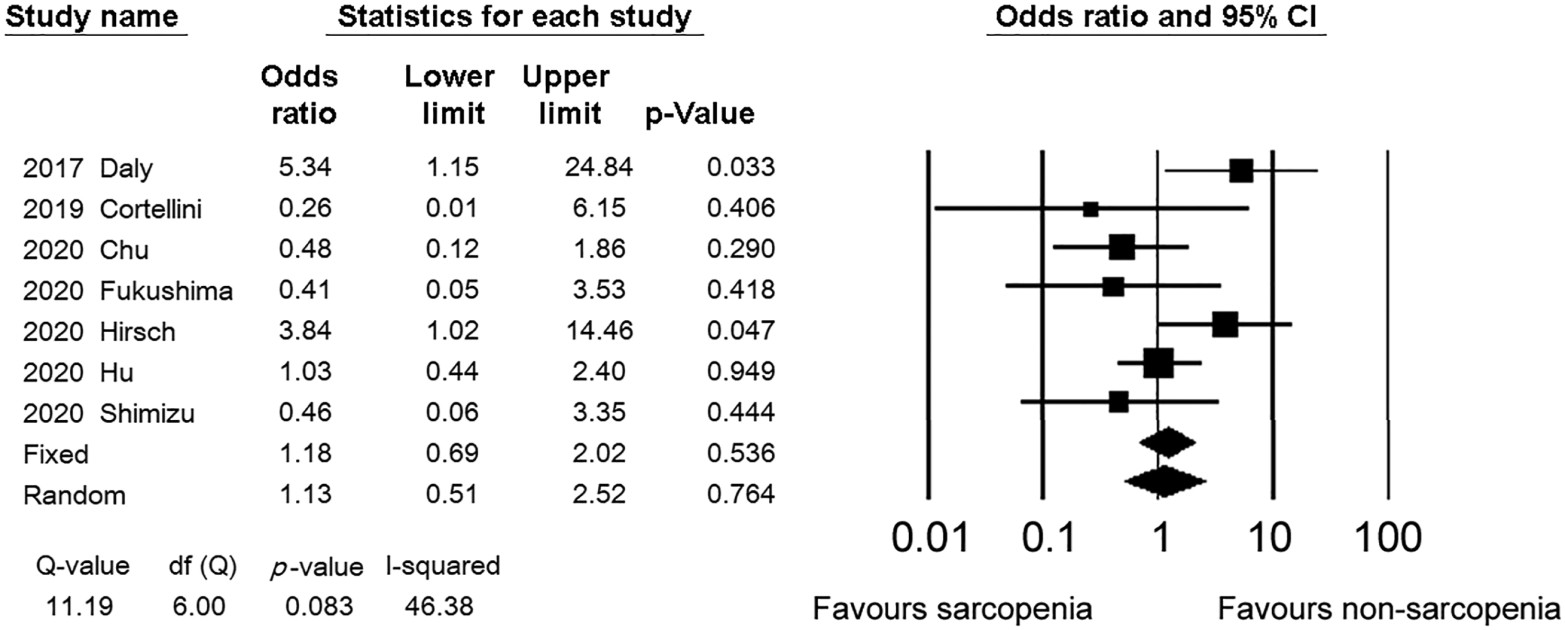
Forest plot showing the odds ratios for severe toxicity between the sarcopenia and non‐sarcopenia patients. The squares represent the hazard ratios for each study. The sizes of the squares and the horizontal lines crossing the squares represent the weight of the study in the random effect model and the 95% confidence intervals, respectively.

The incidence of severe toxicity was assessed in seven studies.^8^, ^11^, ^15^, ^17^, ^19^, ^23^, ^26^ Of them, two performed multivariate analyses^17,19^. The ORs for severe toxicity ranged from 0.26 to 5.34. The pooled OR (95% CI), irrespective of the diagnostic procedure, was 1.13 (0.51–2.52) (*Figure* 4).

### 3.9 Publication bias

**Figure 5:**
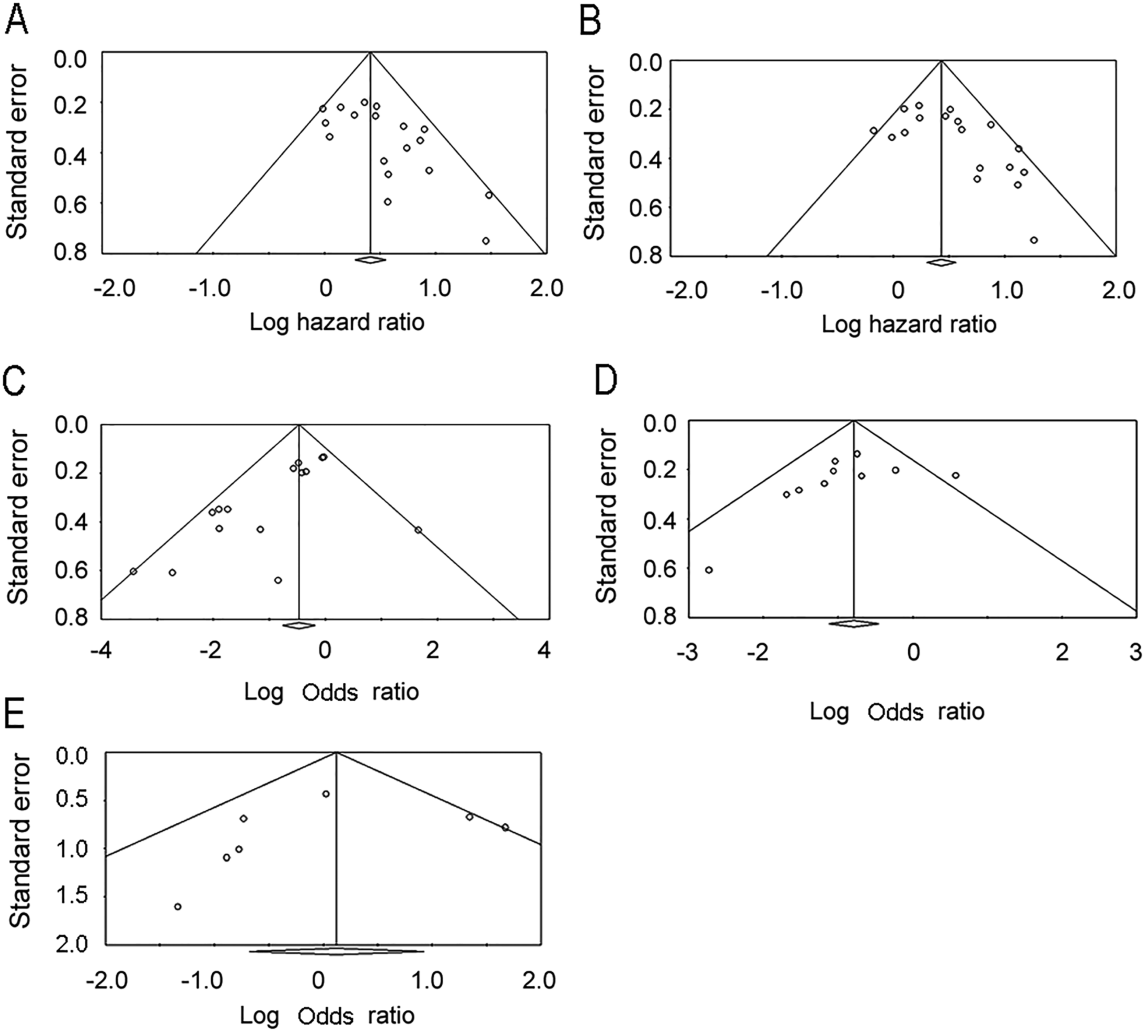
Funnel plot of the hazard ratios for overall survival *(A)* and progression‐free survival *(B)*, and funnel plot of the odds ratio for objective response rate *(C)*, disease control rate *(D)*, and severe toxicity *(E)*.

*Figure* 5 shows funnel plots of the HRs and ORs for the relationship between sarcopenia and OS, PFS, DCR, ORR, and toxicity. These funnel plots showed apparent asymmetry towards higher HRs and asymmetry towards lower ORs. The *P* values derived from the Egger's test of the intercept were 0.006 for OS, 0.013 for PFS, 0.008 for ORR, 0.263 for DCR, and 0.592 for severe toxicity.

## 4 Discussion

In the present study, we found that sarcopenia could predict the response to ICIs and survival after ICI treatment for solid cancers and that its presence was not associated with severe toxicity incidence. The increased mortality observed in the sarcopenia patients was consistent across various cancer types.

Immune checkpoint inhibitors exhibit dramatic and long‐term effects in some patients, while imposing immune‐related adverse events (irAEs) without survival benefits in others. To personalize treatment, facilitate the cost‐effective use of ICIs, and avoid unnecessary irAEs, predictive and prognostic biomarkers have been sought. Some predictive factors for ICI treatment include PDL‐1 expression, haematologic markers, tissue infiltration lymphocytes, metastatic site, inflammatory cytokines, T cell markers, and irAEs.^3^, ^5^, ^41^, ^42^ Sarcopenia has been shown to be a prognostic marker of cancer^7^ and a predictive marker of toxicity during chemotherapy.^43^ A recent meta‐analysis on NSCLC showed that the loss of CT‐defined skeletal muscle mass affected the efficacy of ICIs.^44^ However, the predictive role of sarcopenia in other types of cancer remains to be elucidated. Moreover, although several diagnostic procedures for sarcopenia have been used in the oncologic field, it remains to be elucidated which procedure best predicts the efficacy of ICIs.

Sarcopenia is a muscle disease defined by muscle quantity or quality.^6^ A variety of diagnostic tests and tools are used to detect and diagnose sarcopenia. These include the SARC‐F questionnaire, physical performance tests, muscle strength tests, anthropometric measures, and skeletal muscle measurements.^45^ Among them, muscle measurements using CT, dual‐energy X‐ray, and BIA are popular in the oncology research field. DXA requires special equipment, and the accuracy of BIA is affected by dehydration, which is commonly observed in patients with advanced cancer. In contrast, patients with cancer routinely undergo CT for tumour assessment. Thus, CT is the modality of choice for the diagnosis of sarcopenia in the oncologic field. SMI is the most commonly used index in the literature and is calculated as the total skeletal muscle area at the third lumbar vertebra level divided by the height squared. This index has been shown to be closely correlated with whole body muscle^46^ and is associated with various health‐related outcomes.^6^ PMI is frequently used in research from Japan^14^, ^24^, ^26^, ^28^; it uses the psoas major muscle area instead of the total skeletal muscle area. PMI is easier to calculate, and a cut‐off value has been proposed for Asian adults.^40^ However, some argue that PMI is not a good indicator of sarcopenia.^47^ When PMI and SMI as continuous variables were applied to the same cohort, their HRs for PFS showed comparable values.^17^ Similarly, our meta‐analysis showed that the HRs for OS and PFS were comparable between the two indices, although statistical significance in OS for PMI was not reached owing to the statistical power. Therefore, both SMI and PMI could be used as predictive factors for ICIs.

Previous meta‐analyses on cancer and sarcopenia incorporated only SMI or other muscle mass evaluations as a requirement for inclusion.^7^, ^44^ However, we allowed the inclusion of other methods, such as SMD, muscle mass decrease, and skeletal muscle gauge (SMG). The European consensus statement notes that low muscle quantity or quality is required for the confirmation of sarcopenia diagnoses.^6^ On CT images, the muscle mass area represents muscle quantity, while the muscle density reflects muscle quality. The impairment of muscle quality and infiltration of fat into the skeletal muscle can be indicative of muscle density decrease. SMD is a widely used index for muscle quality and has been shown to be a prognosticator in cancer.^48^ Moreover, SMD, but not SMI, was shown to be associated with physical function,^49^ indicating that it may be a better marker for severe sarcopenia. However, the results of the present meta‐analysis demonstrated that SMD could not predict the survival in patients treated with ICIs. In addition, SMG, an index in which the quantity and quality of skeletal muscle are integrated, was not a predictor of ICI therapy.^29^ Patients with cancer lose weight due to decreased food intake, a catabolic state induced by cancer, and anti‐cancer treatment. Weight loss is a well‐established prognostic factor in patients with cancer.^39^ Similarly, patients with cancer experience loss of skeletal muscle after diagnosis and a decline in gait speed even before diagnosis.^49^ A decrease in skeletal muscle before or during ICI therapy, in other words, the progression of sarcopenia, was associated with adverse outcomes in patients treated with ICIs.^13^, ^17^, ^25^, ^26^ Owing to the small number of studies and differences in the diagnostic procedures, we did not synthesize HRs pertaining to the progression of sarcopenia in the present meta‐analysis. Collectively, of the various sarcopenia measures, muscle mass or its change can be a predictive factor for the efficacy of ICIs.

It may be argued that sarcopenia is reflective of a person's advanced disease status and deteriorated physical condition, resulting in a worse survival. However, our ORR and DCR results suggest that sarcopenia is not a mere prognostic factor but also a predictive factor. Skeletal muscle is known to release myokines, which are muscle‐derived cytokines that exert their effects through the autocrine, paracrine, and endocrine routes.^50^ Among the myokines, interleukin (IL)‐15 increases the proportion of circulating natural killer cells and CD8+ T cells.^51^ More importantly, the administration of IL‐15 in combination with ICIs prolonged the survival of tumour‐bearing mice.^52^ Thus, changes in the myokine levels as a result of sarcopenia may affect the efficacy of ICI treatment, indicating the predictive value of sarcopenia in this therapy.

Skeletal muscle decrease after the initiation of ICIs treatment; that is, PMI and SMI decrease showed higher HRs than pretreatment sarcopenia did (Supporting Information, *Tables* S2 and S4). There are several causes for sarcopenia associated with cancer treatment, which include impaired food intake, reduced activity secondary to fatigue, and a direct effect of drugs on muscle.^53^ Cytotoxic anti‐cancer drugs, including cisplatin, irinotecan, doxorubicin, and etoposide, increase proteolysis through NF‐κB and inflammatory cytokines, resulting in sarcopenia.^53^ Mammalian target of rapamycin (mTOR) is one of the key enzymes involved in the maintenance of skeletal muscle.^54^ Activation of mTOR pathway induces muscle hypertrophy, while blockade of the pathway leads to muscle atrophy.^54^ Everolimus and temsirolimus, mTOR inhibitors used for renal cancer, induced a marked loss of muscle mass in clinical settings.^55^ *In vitro* experiments demonstrated that pembrolizumab activated mTOR pathway.^56^ Therefore, ICIs could affect skeletal muscle directly. Several studies have reported change in skeletal mass after ICIs therapy.^10^, ^17^, ^23^, ^25^, ^26^, ^30^, ^57^ Supporting Information, *Table* S9 summarises the results of these studies. Six out of seven studies assessed skeletal muscle change from 3 weeks to 3 months after baseline and showed reduced muscle mass or muscle attenuation.^10^, ^17^, ^23^, ^25^, ^26^, ^30^ On the contrary, long‐term survivors treated with ICIs showed increased SMI and SMG.^57^ This discrepancy between short‐term and long‐terms might indicate that the direct effect of ICIs on skeletal muscle is minimal and that skeletal muscle loss in short‐term reflects cancer progression and resultant cachexia in non‐responders. Therefore, higher HRs associated with progressive muscle loss could suggest worse survival in non‐responders.

This study has several strengths. First, we investigated a large number of patients using a meta‐analysis. The studies included in the present meta‐analysis were small‐scale retrospective studies. By combining the results, we obtained more reliable estimates of the predictive impact of sarcopenia. Till this date, only one published meta‐analysis has focused on the effect of sarcopenia on ICI efficacy.^44^ However, while the previous meta‐analysis included 576 patients with NSCLC, the present study enrolled 2501 patients with solid cancers, providing a more comprehensive understanding of the predictive ability of sarcopenia. Another strong point is the broad inclusion criteria for muscle measurement. This enabled us to decide which method would be suitable for the prediction of ICI efficacy.

However, our study also has some limitations that must be considered. First, the studies included were of a retrospective nature. A majority of the enrolled studies retrospectively collected patient data. For the precise determination of the response rate and PFS, predefined protocols are mandatory. Second, the methods used for the calculation of the HRs and ORs differed across the studies. Although the use of data from multivariate analyses was desirable, we also included HRs from univariate analyses and estimated HRs from Kaplan–Meier curves. Moreover, the ORs for ORR were adjusted in only one study,^29^ and those for DCR were not adjusted in any of the studies. Even when the HRs were adjusted for confounders, the adjustment was not sufficient owing to the limited number of events. In the investigation of the factors predictive of ICI efficacy, adjustment with established predictive factors, such as PD‐L1 expression or tumour mutation burden, is required. In addition, when investigating the effect of sarcopenia, adjustment with relevant factors, such as body mass index, performance status, and nutritional parameters should be conducted. Third, the cut‐off values associated with the same diagnostic measure varied across the studies. Seven and three cut‐off values were used for PMI and SMI, respectively. The effect of cut‐off values should be investigated using meta‐regression analyses in future studies. Finally, there existed significant publication bias, as shown in *Figure* 5. To reduce the degree of publication bias, we attempted to include non‐English articles. Researchers from non‐English‐speaking countries tend to publish studies of a weaker impact in their local journals and those with positive results in international journals. To retrieve non‐English articles and English articles, we searched Ichushi‐Web, but no Japanese article pertaining to our study topic was identified.

## 5 Conclusions

The number of patients who respond to ICIs is limited. Additionally, ICI treatment imposes a huge financial burden and is associated with irAEs. The identification of responders pre‐therapeutically or in the early phase of the treatment course is critically important. Unfortunately, current companion and complementary diagnostics are insufficient. In the present study, we demonstrated the predictive impact of sarcopenia in patients treated with ICIs. However, sarcopenia alone as a predictor would not be sufficiently useful. Indices comprising the combination of predictive factors are warranted. Further research is required to elaborate on the effective use of ICIs.

## Ethics approval

The approval of the institutional review board was not required because this study was conducted using only previously published data. The authors certify that they comply with the ethical guidelines for publishing in the *Journal of Cachexia, Sarcopenia and Muscle: update 2019*.^58^

## 6 Conflict of interests

There are no conflicts of interest to declare.

## Funding

This work was supported by JSPS KAKENHI Grant Number 19K09868. This work was partially supported by a grant awarded to B Gagnon and M. L. Tremblay from the Terry Fox Research Institute, Canada.

## Author contributions

Y.T. conceived and designed the study and wrote the paper. Y.T. and R.O. collected and analysed the data. N.T., R.O., and H.I. reviewed and revised the manuscript.

7

## Supporting information


**Data S1.** PRISMA Checklist

**Figure S1.** Forest plot showing the prevalence of sarcopenia. The squares represent the hazard ratios for each study. The sizes of the squares and the horizontal lines crossing the squares represent the weight of the study in the random effect model and the 95% confidence intervals, respectively.

**Table S1.** Sensitivity analysis for overall survival
**Table S2.** Hazard ratios for overall survival according to diagnostic measures for sarcopenia
**Table S3.** Sensitivity analysis for progression‐free survival
**Table S4.** Hazard ratios for progression‐free survival according to diagnostic measures for sarcopenia
**Table S5.** Sensitivity analysis for objective response rate
**Table S6.** Odds ratios for objective response rate according to diagnostic measures for sarcopenia
**Table S7.** Sensitivity analysis for disease control rate
**Table S8.** Odds ratios for disease control rate according to diagnostic measures for sarcopenia
**Table S9.** Skeletal muscle change after treatment initiation

## Data Availability

All the data generated during this study are included in this published article and supporting information. All the original data were obtained from the published articles listed in the references.
