# Supplementary figures and images for: Predictive impact of sarcopenia in solid cancers treated with immune checkpoint inhibitors: a meta‐analysis
Source: J Cachexia Sarcopenia Muscle. 2021 Aug 1;12(5):1122–35. doi: 10.1002/jcsm.12755 (PMC8517360; doi:10.1002/jcsm.12755)

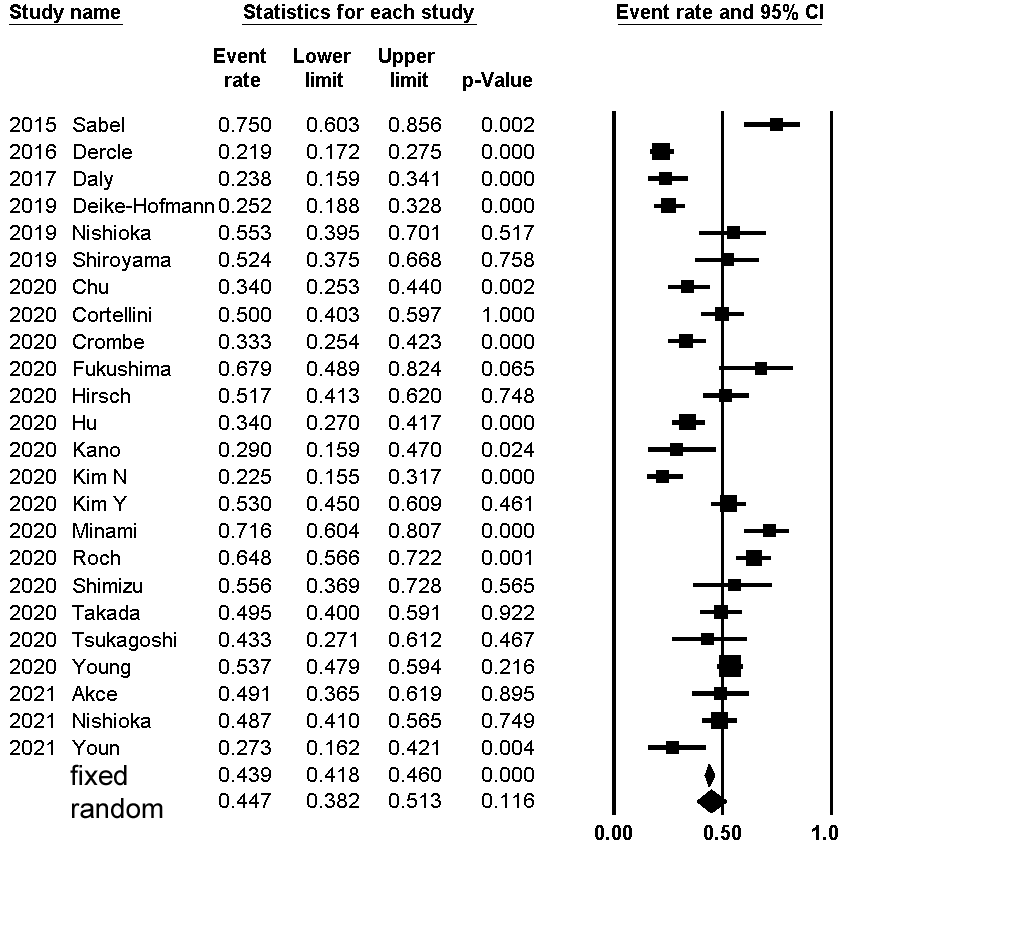

Supplement: Supplementary file 2 — Figure S1. Forest plot showing the prevalence of sarcopenia. The squares represent the hazard ratios for each study. The sizes of the squares and the horizontal lines crossing the squares represent the weight of the study in the random effect model and the 95% confidence intervals, respectively. [file JCSM-12-1122-s002.tif]
